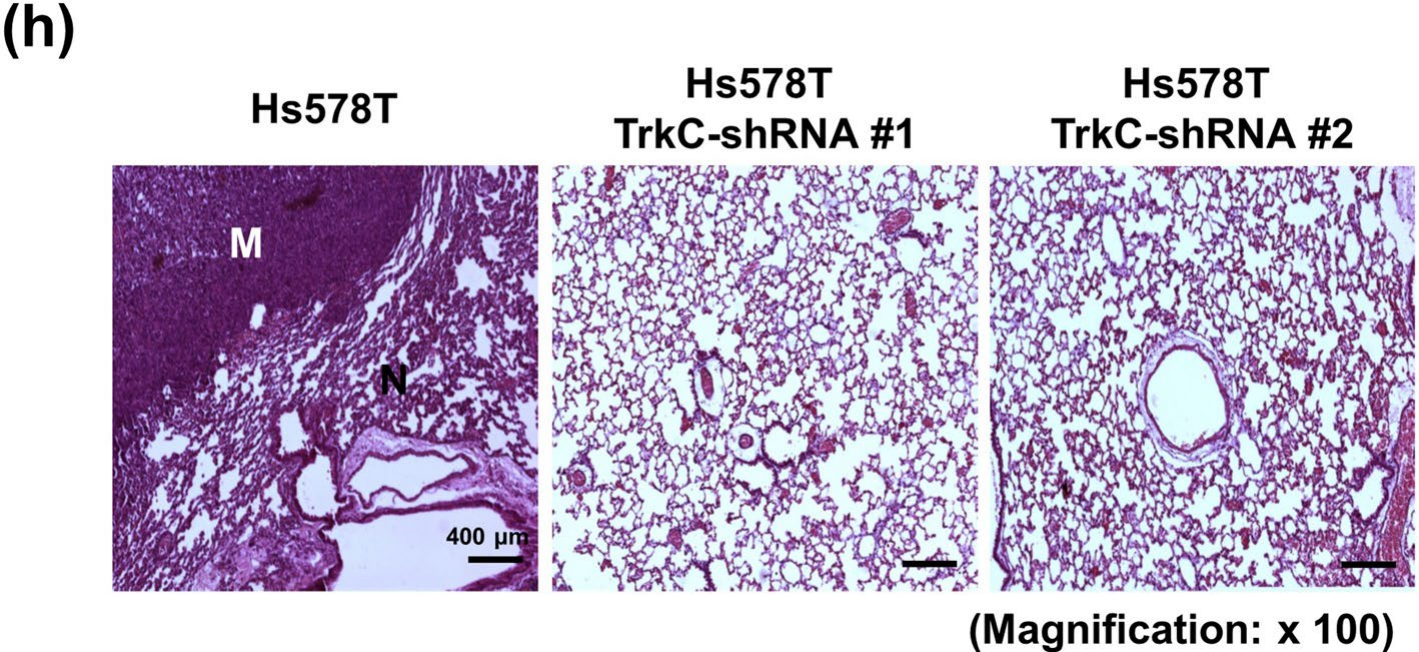# Author Correction: Dysregulated JAK2 expression by TrkC promotes metastasis potential, and EMT program of metastatic breast cancer

**DOI:** 10.1038/s41598-020-68128-6

**Published:** 2020-07-06

**Authors:** Min Soo Kim, Joon Jeong, Jeongbeob Seo, Hae-Suk Kim, Seong-Jin Kim, Wook Jin

**Affiliations:** 1 Laboratory of Molecular Disease and Cell Regulation, Department of Biochemistry, School of Medicine, Gachon University, Incheon, 406-840 Republic of Korea; 2 Department of Surgery, Gangnam Severance Hospital, Yonsei University Medical College, 712 Eonjuro, Gangnam-Gu, Seoul, 135-720 Republic of Korea; 3 Medicinal Chemistry, CMG Pharma, 335, CHA Bio Complex, Pangyo-ro, Bundang-gu, Seongnam-si, Gyeonggi-do 13488 Republic of Korea; 4 TheragenEtex Bio Institute, TheragenEtex Co., Suwon, Gyeonggi-do 16229 Republic of Korea; 5 Nano-Bio Medicine Research Center, Advanced Institutes of Convergence Technology, and Department of Transdisciplinary Studies, Graduate School of Convergence Science and Technology, Seoul National University, Suwon, Kyunggi-do 16229 Republic of Korea; 6 Gachon Medical Research Institute, Gil Medical Center, Incheon, 405-760 Republic of Korea

Correction to: *Scientific Reports* 10.1038/srep33899, published online 22 September 2016

In this Article, Figure 1D contains a duplication: the graphs for the UNC dataset and Curtis dataset are the same. The correct Figure 1D appears below as Figure 1.

Figure 7H also contains a duplication: the images for tumour samples TrkC #1 and #2 are the same. The correct H&E staining panels of Figure 7H appears below as Figure 2.

In the original Article, the methods used for tumour histology were not included. These are given below:

“Tumor histology. Tumor histology was assessed by staining paraffin-embedded tissue sections with hematoxylin and eosin (H&E). Mice were sacrificed, the lungs of each mouse were fixed with 4% paraformaldehyde at room temperature, washed in PBS and then embedded in paraffin. Paraffin-embedded lung sections (5 µm) were stained by standard protocols with hematoxylin (Sigma) for 8 min and with eosin (Sigma) for 1 min. The tissue sections were examined under LSM700 immunofluorescence microscope Z1 (ZEISS) after mounting with Permount mounting medium (Fisher Scientific).”

**Figure:**
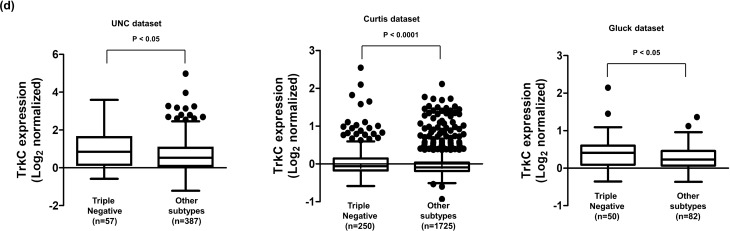

**Figure:**